# Longitudinal fecal amino acid profiles in extremely preterm infants: early-life determinants and associations with late-onset sepsis

**DOI:** 10.1007/s11306-026-02483-9

**Published:** 2026-06-16

**Authors:** A. J. van Wesemael, R. R. de Kroon, N. M. Frerichs, M. M. van Weissenbruch, A. H. van Kaam, N. K. de Boer, E. A. Struys, H. J. Niemarkt, T. G. de Meij

**Affiliations:** 1 Amsterdam Reproduction & Development, Amsterdam UMC, Amsterdam, The Netherlands; 2 Amsterdam Gastroenterology Endocrinology Metabolism Amsterdam, Amsterdam UMC, Amsterdam, The Netherlands; 3 Pediatric Gastroenterology, Emma Children’s Hospital, Amsterdam UMC, Amsterdam, The Netherlands; 4 Neonatal Intensive Care Unit, Emma Children’s Hospital, Amsterdam UMC, Amsterdam, The Netherlands; 5 Department of Gastroenterology and Hepatology, Amsterdam UMC, Amsterdam, The Netherlands; 6 Department of Laboratory Medicine, Amsterdam UMC, Amsterdam, The Netherlands; 7 Neonatal Intensive Care Unit, Máxima MC, Veldhoven, The Netherlands; 8 Department of Electrical Engineering, Technical University Eindhoven, Eindhoven, The Netherlands; 9 Department of Pediatric Gastroenterology, Amsterdam UMC – location AMC, P.O. Box 22660, 1100 DD Amsterdam, The Netherlands

**Keywords:** LC–MS/MS, Amino acids, Metabolomics, Metabolites, Late-onset sepsis, Preterm infants, Probiotics

## Abstract

**Introduction:**

Fecal microbiome and metabolome alterations precede late-onset sepsis (LOS) in preterm infants, but clinical determinants of fecal amino acid (AA) composition and association with LOS development remain poorly understood.

**Objectives:**

This study assessed the early-life determinants of fecal AA composition and AA profiles prior to clinical LOS onset to improve pathophysiological understanding.

**Methods:**

Infants (< 28 weeks’ gestation) with non-staphylococcal LOS were included in a discovery (n = 12) and validation cohort (n = 8), each matched 1:1 to non-LOS controls (n = 20), based on gestational and postnatal age. Using targeted liquid chromatography-tandem mass-spectrometry, this study identified early-life determinants of fecal AAs, analyzing fecal samples collected at week 1 to 4 in controls. Additionally, AA profiles prior to LOS onset were assessed to improve pathophysiological understanding, analyzing samples at t0 and t-3 days from LOS-affected infants and controls.

**Results:**

The most common LOS pathogens were *Escherichia coli* (n = 8), *Serratia* species (n = 4), and *Streptococcus agalactiae* (n = 3). Postnatal age, full-enteral feeding status, probiotic administration, and delivery mode significantly influenced fecal AAs. In the discovery cohort, threonine and glutamine were significantly decreased in affected infants (median [Q1-Q3], threonine: 43.8 [29.3–66.3] vs. 64.5 [48.1–107.3] μmol/L, p = 0.02; glutamine: 19.0 [9.0–38.3] vs. 34.1 [27.0–69.8], p = 0.01), while the validation cohort solely showed a non-significant decrease of threonine. When combining both cohorts, threonine achieved an area under the curve of 0.65 (95%-CI: 0.53–0.76).

**Conclusions:**

These findings suggest several clinical characteristics influence fecal AAs, and altered threonine metabolism may contribute to LOS pathophysiology, warranting evaluation of related metabolites and multi-marker approaches in future studies.

**Graphical Abstract:**

**Figure:**
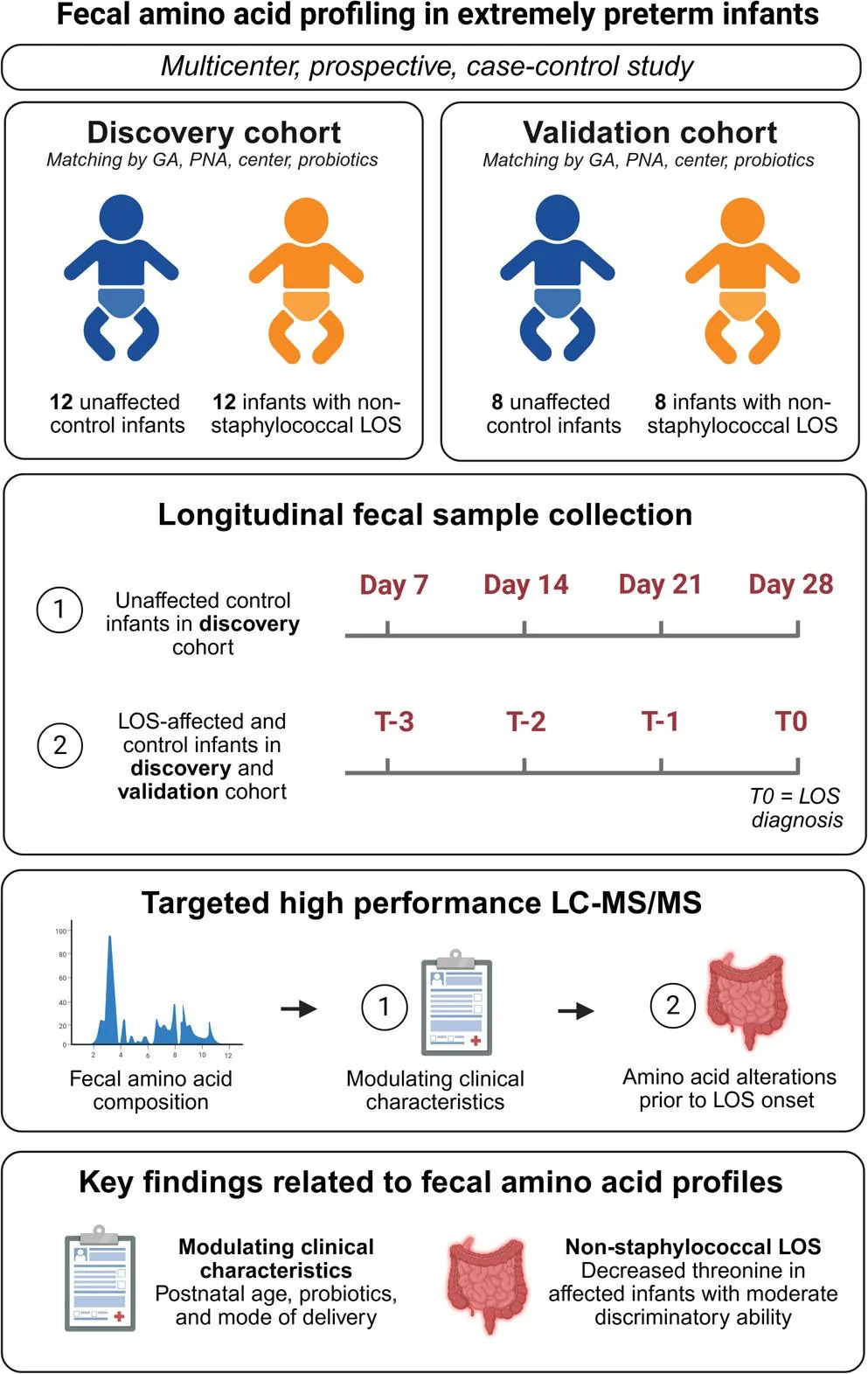

**Supplementary Information:**

The online version contains supplementary material available at https://doi.org/10.1007/s11306-026-02483-9.

## Introduction

Late-onset sepsis (LOS) is a significant cause of mortality and short- and long-term morbidity in preterm infants (Flannery et al., 2022; Tsai et al., 2014). Emerging evidence suggests that the gastrointestinal tract may serve as a reservoir for LOS-associated pathogens (Barcellini et al., 2025; Carl et al., 2014; de Kroon et al., 2025; El Manouni El Hassani et al*.*, 2021; Schwartz et al., 2023; Stewart et al., 2017). High genetic similarity between blood and gut isolates in infants diagnosed with LOS has been demonstrated, supporting the hypothesis of microbial translocation from the gut to bloodstream (Barcellini et al., 2025; Carl et al., 2014). This hypothesis is further strengthened by the increased intestinal abundance of these LOS-causing organisms prior to onset of LOS (de Kroon et al., 2025; El Manouni El Hassani et al*.*, 2021; Stewart et al., 2017). This pattern has been most consistently documented in cases of LOS caused by non-staphylococcal bacteria as opposed to *Staphylococci*, which are commonly associated with catether-associated infections (Cho & Cho, 2019; Pammi et al., 2020). Fecal metabolomic analysis may offer a complementary tool by revealing functional aspects of microbial activity beyond taxonomy (de Kroon et al., 2024; Frerichs et al., 2023, 2025; Liu et al., 2024; Stewart et al., 2017). This may improve our pathophysiological understanding and potentially open a window of opportunity for early disease prediction.

There is a growing interest in the role of the gut metabolome, comprising a wide array of metabolites including amino acids (AAs), in health and disease (Nyangale et al., 2012; Wu et al., 2021). Intestinal AAs are derived from multiple sources, including dietary intake, host secretion, and through both biosynthesis and degradation by intestinal bacteria (Chen & Fang, 2025). AAs are not only absorbed and metabolized by enterocytes, but also available to the gut bacteria, serving as precursors for downstream metabolites. These metabolites can enter the bloodstream and elicit a range of biological functions (Chen & Fang, 2025; Ma & Ma, 2019). Various AAs can promote gut barrier integrity by supporting enterocyte function and tight junctions as well as have immunogenic functions (Ji et al., 2023; Wang et al., 2009). Consequently, AAs represent a key interplay between diet, microbial metabolism, and host physiology, underscoring their importance in understanding gut-associated disease pathophysiology and disease prediction.

However, knowledge on fecal AA concentrations in preterm infants and their association to LOS is sparse. First, the impact of clinical characteristics on preterm fecal AAs is limited. Several studies have described differences in fecal AAs associated with feeding type, probiotics and antibiotics (Bø et al., 2025; Patton et al., 2020; Wang et al., 2025; Ye et al., 2024). However, data on longitudinal AA trajectories is lacking, whereas it is widely accepted that the preterm gut microbiota follows a temporal developmental pattern (Korpela et al., 2018). Secondly, to improve our understanding of the pathophysiology of LOS, a deeper understanding of fecal AA profiles is required. Currently, most studies on disease prediction or disease severity using fecal AAs have focused on colorectal carcinoma and inflammatory bowel disease (Aldars-García et al., 2021; Bosch et al., 2020; Jagt et al., 2022; Vermeer et al., 2025). One study on necrotizing enterocolitis (NEC) showed six fecal AAs to be significantly altered prior to onset of disease with moderate predictive value (Deianova et al., 2022). However, few studies have assessed preclinical fecal metabolome alterations in infants with non-staphylococcal LOS (Liu et al., 2024; Stewart et al., 2017), even though a clear link between gut microbiota and LOS development has been established (Barcellini et al., 2025; Carl et al., 2014; de Kroon et al., 2025; El Manouni El Hassani et al*.*, 2021; Schwartz et al., 2023; Stewart et al., 2017).

In this multicenter, prospective, case–control study, we investigated the longitudinal AA trajectories in the first month of life of extremely preterm infants without NEC or LOS and assessed the impact of clinical characteristics on fecal AA concentrations. Secondly, fecal AAs at the time of diagnosis and in the days preceding non-staphylococcal LOS were assessed and compared to matched control infants to increase our (patho)physiological understanding and evaluate the opportunities of fecal AA-based early risk stratification. To ensure robustness of findings, any preclinical differences in AA concentrations were additionally assessed in a validation cohort.

## Methods

### Participants, matching criteria, and sample selection

This case–control study is embedded within the Generation P study, aimed at developing non-invasive fecal biomarkers for NEC and LOS in extremely preterm infants. Fecal samples are collected daily during the first 29 days of life from all infants with a gestational age < 28 weeks admitted to one of 8 Neonatal Intensive Care Units (NICUs) in the Netherlands. General exclusion criteria are congenital gastrointestinal diseases. This study was approved by the Medical Ethics Review Committee of Amsterdam UMC, location AMC, and by the ethics committees of all participating centers (2014.386, amendment A2020.190). Written informed consent is obtained from the parent(s) or legal guardian(s) for all infants participating in this study.

The database of infants born between March 2021 and December 2023 in 6 participating NICUs was screened for infants with a blood and/or cerebrospinal fluid (CSF) culture-proven non-staphylococcal LOS episode. Infants with gastrointestinal diseases in the first month of life (including NEC Bell’s stage 2A or higher and/or spontaneous intestinal perforation (SIP)) were excluded. At least one fecal sample had to be available for AA analysis, either on the day of clinical onset of LOS (t0), defined as the day the diagnostic LOS work-up was conducted, or in the three days prior (t–3 to t-1). Included LOS-affected infants were 1:1 matched to controls. Infants were eligible as controls if they did not develop culture-proven LOS, NEC, or SIP throughout the study duration. Matching criteria were selected based on their presumed influence on the fecal metabolome (Borrego-Ruiz & Borrego, 2025; Jeong, 2022), and included gestational age (± 5 days), NICU of admittance, probiotics use (yes/no), and postnatal age at sample collection. The decision for this limited set of matching criteria was made a priori to ensure that results would represent a robust, generalizable LOS-associated signal, adequate controls could be identified for every case, and over-matching was avoided given the limited sample size.

First, to identify potential early-life determinants in the first month of life (gestational age, birth weight, delivery mode, antibiotic exposure, probiotic use, and postnatal age), fecal samples from the matched control infants at postnatal day 7 (± 3 days), 14 (± 3 days), 21 (± 3 days) and 28 (± 3 days) were selected. These determinants were selected based on their established roles in shaping preterm microbiome development (Borrego-Ruiz & Borrego, 2025; Jeong, 2022). Next, preclinical samples at t0 and in the three days prior to t0 were selected and analyzed from both LOS-affected infants and control infants, to increase pathophysiological understanding. For example, if t0 of the affected infants occurred on postnatal day 8, the available samples from both the affected infants and matched control were collected and analyzed from day 5 through 8 (t-3 to t0).

Any fecal AAs that were significantly altered between affected infants with non-staphylococcal LOS and control infants were further analyzed in a validation cohort. For the validation cohort, the database of infants born between January 2024 and December 2024 in 7 participating NICUs was screened for eligible participants, and the same in- and exclusion criteria and matching criteria were employed as described above. No additional matching criteria were included based on found early-life determinants in the discovery cohort, in order to accurately assess the reproducibility and generalizability of the observed associations in a validation cohort under comparable conditions.

### Clinical data and sample collection

Clinical data from the first 29 days of life were extracted from electronic patient files using Castor EDC. The collected data included demographic data (e.g. gestational age, birthweight, mode of delivery) and clinical data (e.g. probiotic or antibiotic administration, disease occurrence, and feeding practices). FEF was defined as the cessation of both parenteral feeding and iv glucose. Fecal samples were collected daily from diapers by health care professionals on the NICU and stored in sterile fecal sample containers at −20 °C within 1 h of collection. For long-term storage, samples were stored at −80 °C.

### Fecal targeted amino acid analysis

First, lyophilization was conducted to reduce the risk of potential bias caused by differences in fecal water content. Fecal samples were thawed and homogenized to ensure uniform consistency. 1 ml of sterile demineralized water was added to fecal aliquots. The solution was homogenized by vortexing at room temperature. Prior to lyophilization, the fecal solution was rapidly frozen by emersion of the freeze drying flask with the sample in dry ice with isopropanol. Afterwards, the sample was subjected to lyophilization (laboratory lyophilizer type 2, Zirbus Technology Benelux B.V.). Drying was performed at −80 °C under a vacuum of 0.090 mbar for at least 8 h. Lyophilized samples were then transferred to a sterile tube, sealed, and stored at −20 degrees until further processing. For the targeted AA analysis*,* lyophilized samples were rehydrated with demineralized water to a concentration of 100 mg/5000 μl and the dilution was homogenized by vortexing at room temperature. Next, fecal AAs were quantified using targeted high-performance liquid chromatography-tandem mass spectrometry (LC–MS/MS), based on a protocol described previously (Opperman et al., 2025). AAs were quantified using commercially available mixtures of stable isotope-labelled internal standards and calibrators (Chromsystems Instruments & Chemicals GmbH, Germany). Concentrations were calculated using single point calibration based on analyte to internal standard response ratios. The selection of measured AAs was based on a historical selection from a previous study (Bosch et al., 2018). The chromatographic and mass spectrometric parameters and quantification limits for each amino acid are described in Table S1.

### Statistical analysis

All statistical analysis and visualizations were done using R (version 4.4.3). For normally distributed continuous data (determined by visually inspecting histograms), the student t-test was applied and data were presented as mean and standard deviation. For non-normally distributed, the Mann–Whitney U test was applied and data were presented as median and interquartile range (IQR). Categorical data were analyzed using Chi-squared test or Fisher's exact test.

For the AA analyses, we assessed the impact of selected clinical characteristics (gestational age, birth weight, delivery mode, postnatal age, FEF status (achieved FEF or did not achieve FEF prior to sampling), antibiotic exposure, and probiotic use, respectively) on fecal AA profiles in the matched control infants. Using the lmer function (lme4 package), linear mixed-effects modeling was applied, accounting for repeated measurements of the same infant, to assess the impact of postnatal age on each individual AA concentration. Next, linear mixed-effects modeling, accounting for both repeated infant measurements as well as postnatal age, was applied to assess the impact of the other clinical characteristics. Metabolome β-diversity analysis was performed using Principle Component Analysis (PCA) based on Euclidean distances (after autoscaling metabolite concentrations) to evaluate the influence of the clinical characteristics on sample clustering. This was statistically evaluated using Permutational Multivariate Analysis of Variance (PERMANOVA; adonis2 function; vegan package) based on 9,999 permutations. Each factor was tested separately to estimate the proportion of variance explained (R^2^) and associated significance (p-value < 0.05).

Secondly, we compared the AA composition of the preclinical fecal samples of the LOS-affected infants and the matched controls using the Mann–Whitney U-test, followed by false discovery rate (FDR) correction with Benjamini Hochberg method. As this was an exploratory study, non-adjusted significant alterations between affected infants and controls were also reported and interpreted with caution. AAs that showed statistically significant alteration were subsequently validated in an independent validation cohort to confirm robustness of the findings. To evaluate the discriminatory potential of these candidate AAs, receiver operating characteristic (ROC) curve analysis was performed on preclinical samples of both the discovery and the validation cohort (pROC package), and the predictive performance of each AA was quantified using the area-under-the-curve (AUC).

## Results

### Baseline characteristics

For the discovery cohort, 370 infants were screened. 107 infants were diagnosed with at least one culture-proven LOS episode. Of these infants, 12 were eligible for inclusion in the current study and 1:1 matched to control infants (93 fecal samples). Table S2 shows an overview of included samples per timepoint and subgroup. The clinical characteristics were similar between the two groups, apart from total antibiotic and probiotic exposure, as shown in Table 1. However, antibiotic and probiotic exposure prior to LOS onset was similar. For the validation cohort, a total of 200 infants were screened. Out of 49 infants diagnosed with culture-proven LOS, 8 infants could be included in the validation cohort and were 1:1 matched to control infants (38 fecal samples) (Table S2). Demographic characteristics of the LOS-affected infants and matched controls in the validation cohort are displayed in Table S3. Apart from a lower proportion of vaginally born infants in the validation cohort, there were no major differences between the affected infants in the discovery cohort and validation cohort (Table S4). *Escherichia coli* was the most common causative pathogen in both the discovery and the validation cohort (Table S5).

**Table 1:** Demographic and clinical characteristics of infants included in the discovery cohort

	Non-staphylococcal LOS (n = 12)	Control infants (n = 12)	p-value
**Gestational age, median (weeks + days), IQR (days)**	26 + 2 (13)	26 + 3 (12)	0.862
**Birth weight (grams), median (IQR)**	825 (196)	923 (338)	0.544
**Biological sex, female, n (%)**	10 (83)	7 (58)	0.369
**Mode of delivery, vaginal delivery, n (%)**	9 (75)	9 (75)	1.000
**Apgar score < 7 at 5 min, yes, n (%)**	1 (8)	4 (33)	0.107
**Cumulative days iv antibiotics in first month of life, median (IQR)**	12 (17)	4 (4)	**0.002**
**Ratio cumulative days iv antibiotics/admission days in first month of life, median (IQR)**	0.50 (0.37)	0.14 (0.12)	** < 0.001**
**Cumulative days iv antibiotics prior to onset t-1**^**1**^**, median (IQR)**	3 (3)	3 (1)	0.402
**Time to full enteral feeds (days), median (IQR)**	10 (2)	9 (2)	0.060
**Volume feeds (ml/kg/day) at full enteral feeding, median (IQR)**	159 (6)	160 (15)	0.454
**Reached full enteral feeding**^**2**^** at t-1, yes, n (%)**	6 (50)	6 (50)	1.000
**Volume feeds (ml/kg/day) at t-1, median (IQR)**	116 (127)	119 (102)	0.622
**Enteral feeding type at t-1:** -Mother’s own milk, n (%) -Donor human milk, n (%) Human milk^3^, n (%)	7 (58) 2 (17) 3 (25)	6 (42) 3 (25) 3 (25)	0.065
**Received enteral probiotics in the first 29 days of life**^**4**^**, yes, n (%)**	9 (75)	9 (75)	1.000
**Cumulative days enteral probiotics in first month of life, median (IQR)**	5 (17)	26 (8)	**0.034**
**Ratio cumulative days enteral probiotics/admission days in first 29 days of life, median (IQR)**	0.57 (0.52)	0.90 (0.28)	**0.034**
**Cumulative days enteral probiotics prior to t-1, median (IQR)**	3 (5)	4 (4)	0.659
**Postnatal age (days) at clinical onset of LOS**^**5**^**, median (IQR)**	8 (9.5)	n.a	n.a

^1^t-1 is the defined as the day prior to diagnostic work-up that resulted in LOS diagnosis (t0). ^2^Full enteral feeding is defined as the cessation of both parenteral feeding and iv glucose. ^3^Not specified in clinical files whether it was donor human milk or mother’s own milk. ^4^The probiotics mixture administered to the included infants is ProPrems©, according to the Dutch national guidelines. ^5^Clinical onset of LOS is defined as the day the diagnostic work-up is conducted that resulted in LOS diagnosis, which included at least a blood culture. A p-value < 0.05 was considered significant and were presented in bold. * IQR *interquartile range*; LOS *late-onset sepsis*; n.a. *not applicable

### Postnatal age, probiotic exposure, delivery mode, and full enteral feeding status influence fecal amino acid concentrations in infants without NEC or sepsis

**Fig. 1:**
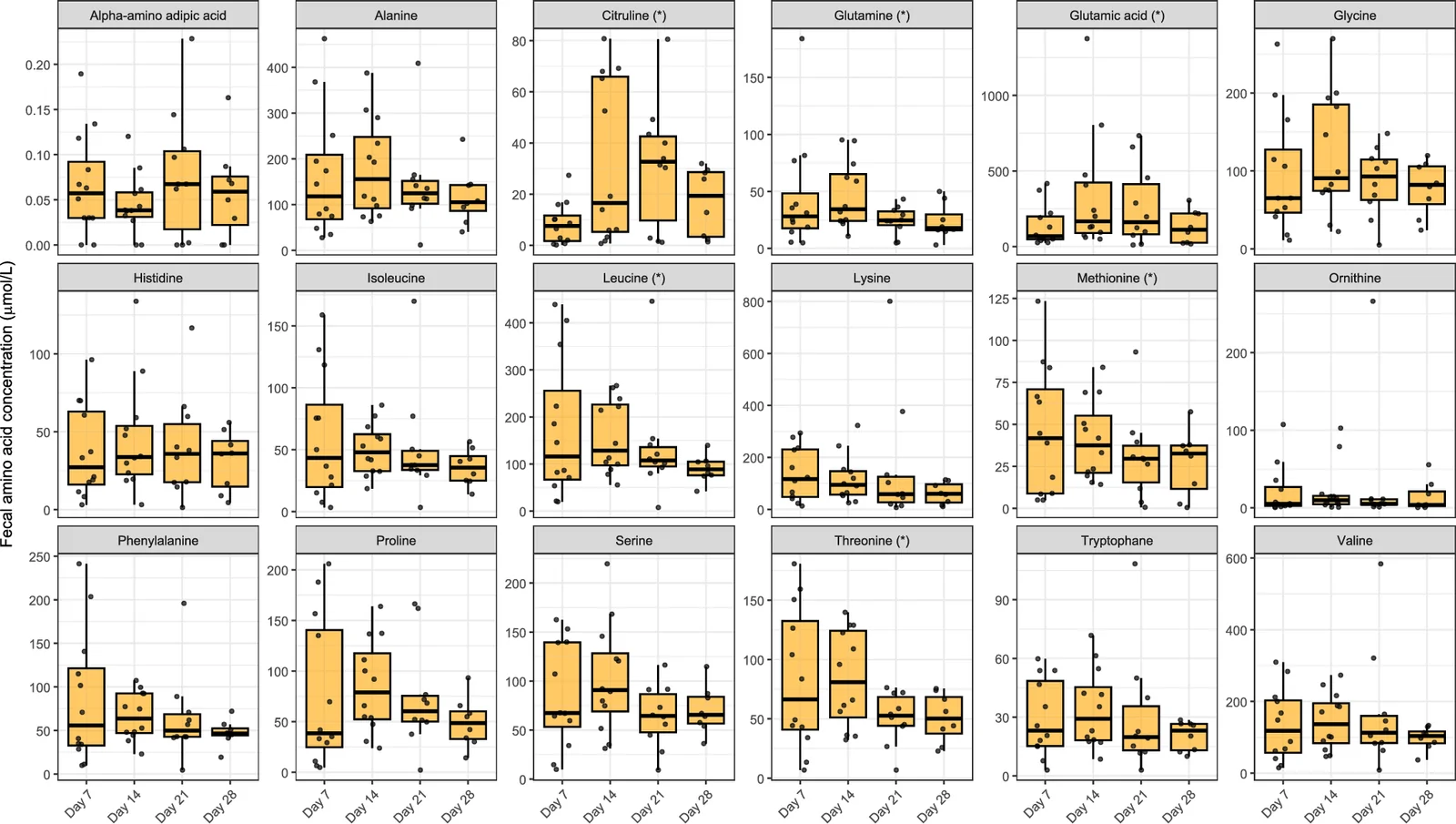
Amino acid concentrations (μmol/L) in the first month of life in control infants without necrotizing enterocolitis and/or sepsis. The figure displays the temporal trajectories of the fecal AA concentration during the first month of life in unaffected matched control infants from the discovery cohort (n = 12 infants). Each panel depicts one of the measured AAs in infants without NEC and/or sepsis. The y-axis shows the fecal AA concentration (μmol/L), and the x-axis shows the postnatal age grouped into sampling windows (day 7 ± 3 days, day 14 ± 3 days, day 21 ± 3 and day 28 ± 3 days). In each boxplot, the horizontal line represents the median AA concentration per sampling window, the box the IQR (25th–75th percentile), and the whiskers extend to the most extreme values within 1.5 × IQR. Linear mixed-effects models accounting for repeated measurements within control infants were used to evaluate the effect of postnatal age on individual AA concentrations. A p-value < 0.05 was considered statistically significant, which is expressed by an asterisk in the header of the plot. AA, amino acid; IQR, interquartile range; NEC, necrotizing enterocolitis

**Fig. 2:**
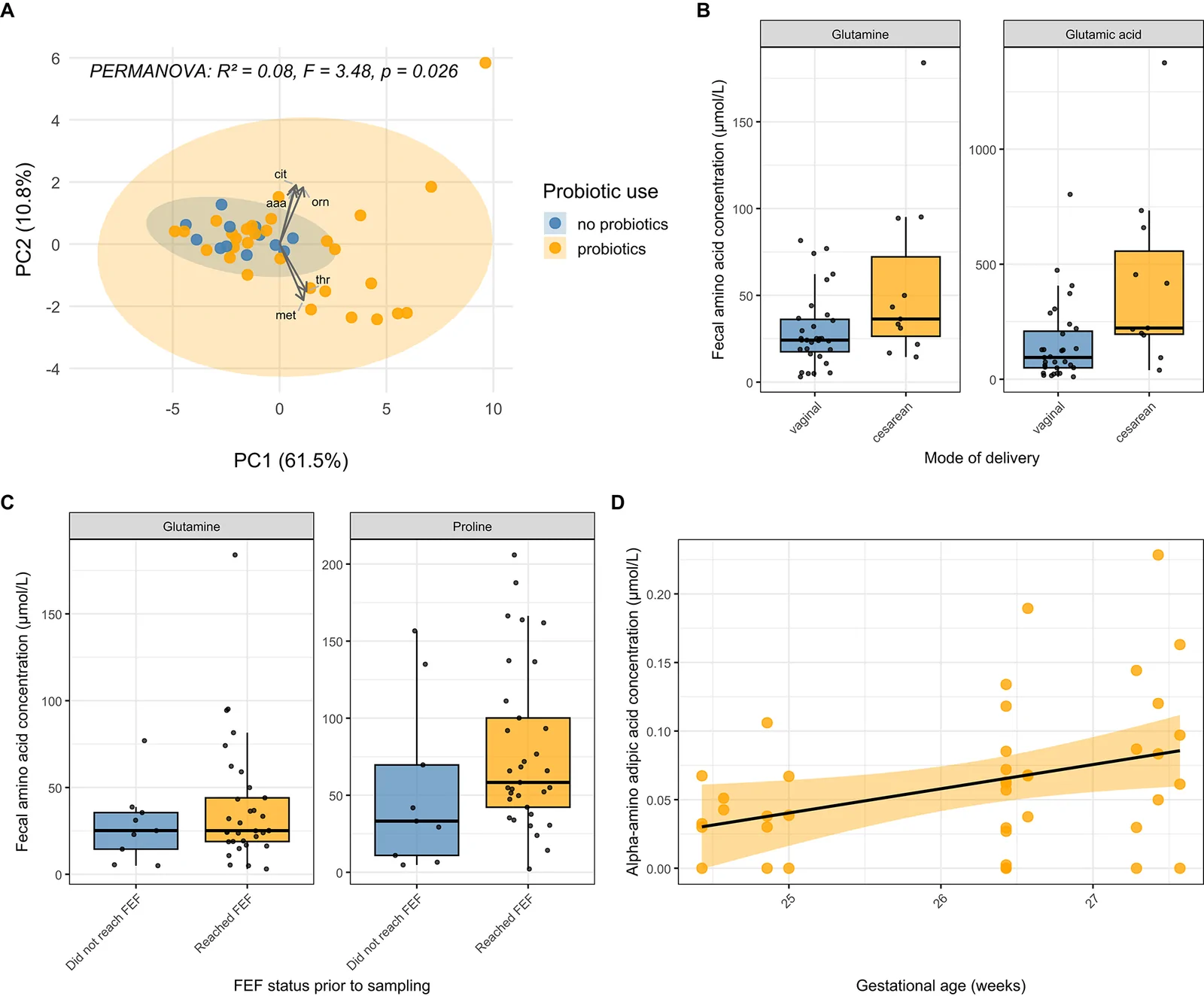
Associations of fecal amino acid concentrations (μmol/L) with probiotic administration, mode of delivery, full enteral feeding, and gestational age. All associations were assessed in the matched control infants, without NEC and sepsis, of the discovery cohort (12 infants, 42 fecal samples). **A** displays a PCA, based on Euclidean distance, of the metabolomic profiles of probiotic-naïve (n = 3 infants, yellow) and probiotic-supplemented control infants (n = 9 infants, blue). The x- and y-axes represent the first and second PCs (PC1: 61.5%, PC2: 10.8%); arrows indicate the five AAs contributing most strongly to sample ordination (methionine, threonine, ornithine, citrulline, and α-amino adipic acid), with arrow length reflecting the relative strength of each AAs contribution. Statistical evaluation was done using PERMANOVA based on 9,999 permutations with a p-value < 0.05 considered significant. **B** shows the fecal AA concentration for glutamine and glutamic acid, stratified by mode of delivery (vaginal delivery (n = 9 infants) and cesarean section (n = 3 infants), respectively). **C** shows the fecal AA concentration for glutamine and proline, stratified by FEF status (did not reach FEF prior to sampling (9 samples) and reached FEF prior to sampling (33 samples), respectively). In each boxplot, the horizontal line represents the median AA concentration per subgroup, the box the IQR (25th–75th percentile), and the whiskers extend to the most extreme values within 1.5 × IQR. The dots represent individual samples. **D** shows a scatterplot of the concentration of α-amino adipic acid on the y-axis, with gestational age in weeks on the x-axis. The dots represent individual fecal samples; the line represents the fitted linear regression that demonstrates the estimated mean trend of the AA concentration. The shaded grey area is the 95% confidence interval of the estimated mean. Linear mixed-effects models, accounting for repeated measurements within the control infants and postnatal age, were used to evaluate the effect of mode of delivery (**B**), FEF status (**C**) and gestational age (**D**) on the fecal AA concentration. aaa, alpha-amino adipic acid; AA, amino acid; cit, citrulline; FEF, full enteral feeding; IQR, interquartile range; met, methionine; NEC, necrotizing enterocolitis; orn, ornithine; PC, principal component; PCA, principal component analysis; PERMANOVA, permutational multivariate analysis of variance; thr, threonine

The temporal fecal AA profiles of the matched control infants in the first month of life (n = 12 infants; 42 fecal samples) are displayed in Figure 1 (medians and IQR) and Supplemental Figure S1 (estimated marginal means). Linear mixed-effects modeling, with patient identification as random effects, revealed a significant decrease in fecal concentrations of glutamine (*β* = −8.88, SE = 4.21, p = 0.04), threonine (*β* = −13.20, SE = 4.86, p = 0.01), methionine (*β* = −6.35, SE = 2.91, p = 0.04) and leucine (*β* = −27.05, SE = 12.83, p = 0.04). A linear trajectory in AA concentrations over the first month of life cannot be assumed for all AAs. Therefore, the linear mixed-effects model was refitted with postnatal age as a categorical variable (e.g. sampling at day 7, 14, 21, and 28), with day 7 as a reference category. In this model, additional statistically significant effects of postnatal age were seen for citrulline at day 14 and 21 (*β* = 23.71, SE = 7.80, p = 0.005; and *β* = 23.3, SE = 8.24, p = 0.008) and glutamic acid at day 14 (*β* = 198.25, SE = 82.73, p = 0.02), while no significant postnatal age-specific differences were seen for glutamine and methionine. Next, we assessed the effect of selected clinical characteristics on fecal AAs. Using PCA based on Euclidean distances of all quantified AAs, we found significant overall effects of probiotic exposure (R^2^: 0.08; F-value: 3.48; p-value: 0.026), and patient ID (R^2^: 0.48; F-value: 2.55; p-value < 0.001) on the metabolite profiles. Figure 2A shows the ordination of samples colored by probiotic exposure, with the top five AAs that contribute most to the ordination of samples. Next, we performed a linear mixed-effects model to assess the impact of each parameter on specific AAs, correcting for postnatal age and including patient identification as a random effect. It revealed no statistically significant AA concentrations associated with probiotic exposure. For the other postnatal factors, it revealed infants delivered by cesarean section (C-section) showed higher fecal glutamic acid (*β* = 253.3 ± 108.1, p = 0.041) and glutamine (*β* = 26.9 ± 11.2, p = 0.039) compared to vaginal delivery (Fig. 2B), and samples collected after reaching FEF showed higher fecal proline (*β* = 57.1 ± 19.1, p = 0.006) and glutamine (*β* = 40.3 ± 13.4, p = 0.005) (Fig. 2C). Additionally, gestational age was associated with increased α-amino adipic acid, although concentrations were low (*β* = 0.003 ± 0.001, p = 0.023) (Fig. 2D).

### Preclinical amino acid alterations in preterm infants with non-staphylococcal late-onset sepsis

**Fig. 3:**
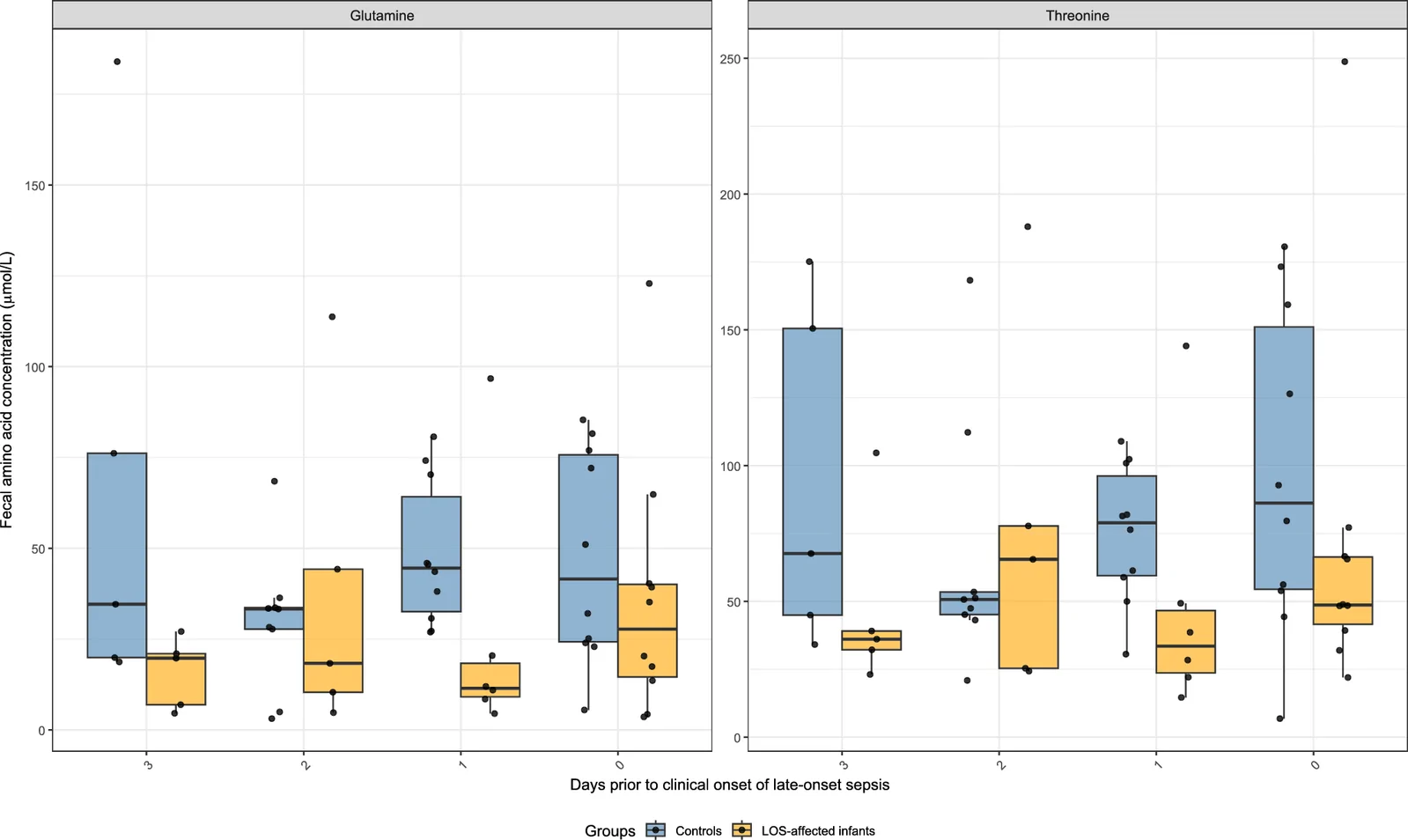
Temporal course of glutamine and threonine concentrations (μmol/L) in infants diagnosed with non-staphylococcal late-onset sepsis and matched controls on day of diagnosis and three preceding days. The figure displays the glutamine and threonine concentration stratified by subgroup (control infants in blue vs. LOS-affected infants in orange) and per day (t-3 to t0) in the discovery cohort (12 vs. 12 infants, 26 vs. 34 samples). The central line of each boxplot represents the median, the box edges correspond to the 25th and 75th percentiles (IQR), and the whiskers extend to the most extreme values within 1.5 × IQR. Individual points beyond the whiskers are plotted as outlier. AA, amino acid; IQR, interquartile range; LOS, late-onset sepsis

**Fig. 4:**
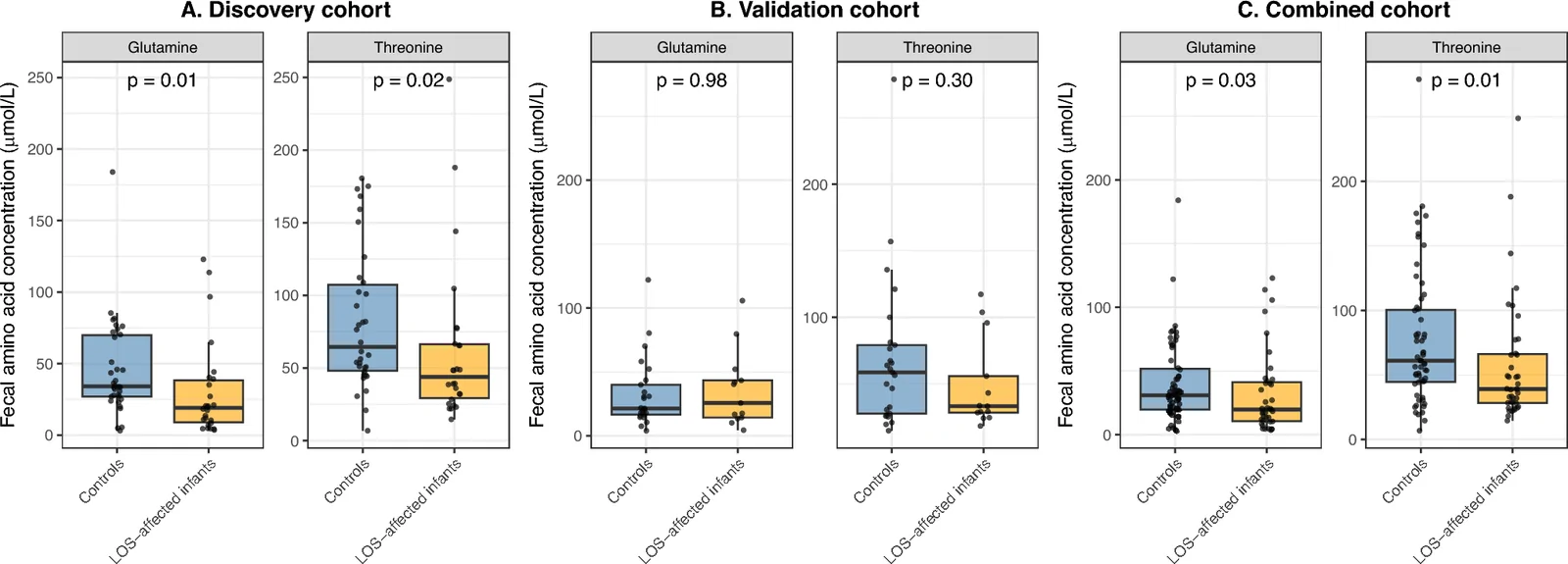
Glutamine and threonine concentrations (μmol/L) in infants diagnosed with non-staphylococcal late-onset sepsis and matched controls in the discovery cohort, validation cohort, and combined cohort. The figure displays the glutamine and threonine concentration stratified by subgroup (control infants vs. LOS-affected infants) in the discovery cohort (**A**, 12 vs. 12 infants, 26 vs. 34 samples), validation cohort (**B**, 8 vs. 8 infants, 13 vs. 25 samples), and group cohort (**C**, 20 vs. 20 infants, 39 vs. 59 samples). The central line of each boxplot represents the median, the box edges correspond to the 25th and 75th percentiles (IQR), and the whiskers extend to the most extreme values within 1.5 × IQR. Individual points beyond the whiskers are plotted as outliers. Statistical comparisons between groups were performed using the Mann–Whitney U test, with p-values indicated on the plots. A p-value < 0.05 was considered significant. AA, amino acid; IQR, interquartile range; LOS, late-onset sepsis

We next compared the fecal AA profiles of LOS-affected infants up to three days before diagnosis to matched control samples. Prior to FDR correction in the discovery cohort (n = 24 infants), both glutamine and threonine concentrations were significantly decreased in LOS-affected infants vs. controls (glutamine, median ± IQR, 19.0 ± 29.3 vs. 34.1 ± 42.8, p = 0.01; threonine, 43.8 ± 37.1 vs. 64.5 ± 59.2, p = 0.02, Table 2, Fig. 3 and 4A). After correction for multiple testing, none of the AAs between affected infants and controls significantly differed. When assessing the validation cohort (n = 16 infants) separately from the discovery cohort, there were no significant differences between the two subgroups for glutamine and threonine concentrations, though threonine still showed a non-significant decrease in LOS-affected infants vs. controls (glutamine, 25.7 ± 29.1 vs. 21.3 ± 23.3, p = 0.98; threonine, 33.1 ± 27.5 vs. 58.5 ± 51.4, p = 0.30, Fig. 4B). When combining both cohorts (n = 40 infants), both threonine and glutamine were significantly decreased in the LOS-affected infants (threonine, 39.1 ± 37.8 in affected infants vs. 61.1 ± 55.8 in controls, p = 0.01; glutamine, 19.7 ± 30.5 vs. 30.9 ± 31.9, p = 0.03, Fig. 4C). Univariate ROC curve analysis of the combined cohort demonstrated an AUC of 0.65 (95%- confidence interval (CI): 0.53–0.76) with optimal cut-off value at 49.5 μmol/L for threonine. The sensitivity was 70% and specificity 70%. For glutamine, the AUC was 0.63 (95%-CI: 0.51–0.75), with optimal cut-off value at 21.2 μmol/L and a sensitivity of 59% and specificity of 71%.

**Table 2:** Amino acid concentrations (μmol/L) in infants with non-staphylococcal late-onset sepsis and their matched controls

Type	Name	Control infants (n = 12 infants; 26 samples)		Non-staphylococcal LOS (n = 12 infants; 34 samples)		P-value	Adjusted P-value
		Median (µmol/L)	IQR (µmol/L)	Median (µmol/L)	IQR (µmol/L)		
*Conditionally essential amino acid*	Glutamine	34.1	42.8	19.0	29.3	0.01*	0.12
	Proline	39.7	74.5	42.4	51.1	0.48	0.93
	Glycine	77.1	46.4	74.0	77.9	0.75	0.93
	Serine	73.2	39.9	59.5	57.3	0.10	0.43
*Essential amino acid*	Tryptophan	25.6	27.9	22.0	19.8	0.22	0.58
	Histidine	32.6	31.8	25.9	46.0	0.56	0.93
	Methionine	35.3	36.6	31.0	32.5	0.85	0.93
	Isoleucine	37.5	32.9	40.4	56.7	0.85	0.93
	Phenylalanine	54.0	36.4	51.5	70.8	0.75	0.93
	Threonine	64.5	59.2	43.8	37.1	0.02*	0.15
	Lysine	82.2	90.0	56.9	88.5	0.18	0.55
	Valine	103.7	83.4	103.9	144.5	0.88	0.93
	Leucine	117.8	92.5	116.4	124.9	0.44	0.93
*Non-essential proteogenic amino acid*	Glutamic acid	106.2	224.2	62.0	187.4	0.09	0.43
	Alanine	124.2	80.6	134.6	153.6	0.98	0.98
*Non-proteogenic amino acids*	Citrulline	7.5	27.8	2.4	10.8	0.13	0.48
	Ornithine	6.3	12.9	5.0	13.7	0.71	0.93
	Alpha-amino adipic acid	0.1	0.0	0.1	0.1	0.71	0.93

The table displays the median and IQR for each of the measured AAs in the control infants (12 infants, 26 samples) and the infants with non-staphylococcal LOS (12 infants, 34 samples) in the discovery cohort. For the comparison, all samples from 3 days prior to diagnosis to day of diagnosis (t-3 to t0) are pooled per subgroup. The measured AAs were classified into four groups: conditionally essential, essential, non-essential, and non-proteogenic AAs. Affected infants and matched controls were compared using the Mann–Whitney U-test, followed by False Discovery Rate correction with Benjamini Hochberg method, to account for multiple comparisons. A p-value < 0.05 was considered significant and were presented in bold. *AA *amino acid*; IQR *interquartile range*; LOS *late-onset sepsis

## Discussion

In this multicenter case–control study, we investigated longitudinal fecal AA profiles of extremely preterm infants to assess their physiological trajectory and modulating clinical characteristics in the first month of life. We demonstrated longitudinal changes in the first month of life for several AAs, as well as identified probiotic exposure, delivery mode and FEF status as influencing factors on fecal AA concentrations. Additionally, we assessed AA concentrations prior to onset of non-staphylococcal LOS, to enhance pathophysiological understanding, and found decreased threonine concentrations with moderate discriminatory ability.

First, we demonstrated significant changes in the first month of life in glutamine, threonine, methionine and leucine (decrease), as well as citrulline and glutamic acid (increase) in control infants. These findings indicate dynamic maturation patterns, in line with previous studies demonstrating postnatal age as a major driver of the gut microbiota and urinary AA changes (Astono et al., 2024). Other influencing factors were probiotic administration, FEF status and mode of delivery, consistent with findings in microbiome research (Collado et al., 2015). Probiotic administration induced shifts in overall fecal AA composition, rather than significant changes in any single AA. The administrated probiotic supplement (ProPrems©) consists of a mixture of *Bifidobacterium longum* subspecies *infantis, Bifidobacterium animalis* subspecies *lactis*, and *Streptococcus thermophilus.* While carbohydrate fermentation dominates in Bifidobacteria, several AAs are co-metabolized by these bacteria (Devika & Raman, 2019; Ferrario et al., 2015). Moreover, studies in human milk demonstrate that *Bifidobacteria* can also produce amino-acid derived metabolites, including aromatic lactic acids (derived from tryptophan, phenylalanine, tyrosine), known to accumulate in infant feces and exert immunomodulatory roles (Laursen et al., 2021). These studies support the altered fecal AA composition identified in probiotic-exposed infants. Additionally, FEF-status was associated with increased glutamine and proline. Both of these AAs, and glutamic acid, the precursor to glutamine, are present in high concentrations in human milk, potentially explaining the higher fecal availability (Zhang et al., 2013). Lastly, we demonstrated increased glutamic acid and glutamine in infants born via C-section, which typically lack the maternal vaginal and fecal seeding that drives early colonization with *Bifidobacterium* and *Bacteroides.* Instead, *Staphylococci* are known to dominate the gut microbiome of infants born via C-section (Rutayisire et al., 2016). *Bifidobacteria* and *Bacteroides* consume glutamic acid for biosynthesis, and convert them into downstream metabolites (Schimmel et al., 2023). The lack of *Bifidobacteria* and *Bacteroides* in infants born through C-section may explain the increased glutamic acid. However, the present study is limited to fecal AAs and does not include microbiome composition. No associations between microbial taxa and AA concentrations could be made.

Next, we sought to identify specific AAs associated with non-staphylococcal LOS up to 3 days prior to onset of symptoms. We solely selected infants with LOS due to non-staphylococcal species, as staphylococcal LOS is frequently associated with catheter-derived infection rather than an origin in the gut (Jansen et al., 2024). Through our matching procedures, the confounding effects of postnatal age, NICU of admittance and probiotics were minimized. In the discovery cohort, decreased threonine and glutamine was associated with the development of LOS. To validate our findings, we analyzed a separate validation cohort, in which only threonine showed the same direction of effect, though non-significant. Univariate ROC curve analyses of the combined cohort (discovery and validation combined) demonstrated a moderate AUC of 0.65 (95%-CI: 0.53–0.76) for threonine, indicating that this single parameter offers suboptimal discriminatory ability for accurately identifying at-risk infants. The moderate discriminatory accuracy may be explained by the varying LOS pathogen distribution between the discovery and validation cohort, possible residual confounding by factors that influence AA patterns, and the small cohort size, preventing pathogen-specific subgroup analysis. Since AA metabolism varies substantially between species (Blachier, 2023; Enany et al., 2023), this may consequently affect fecal profiles and hamper the identification of general LOS-specific AAs.

**Fig. 5:**
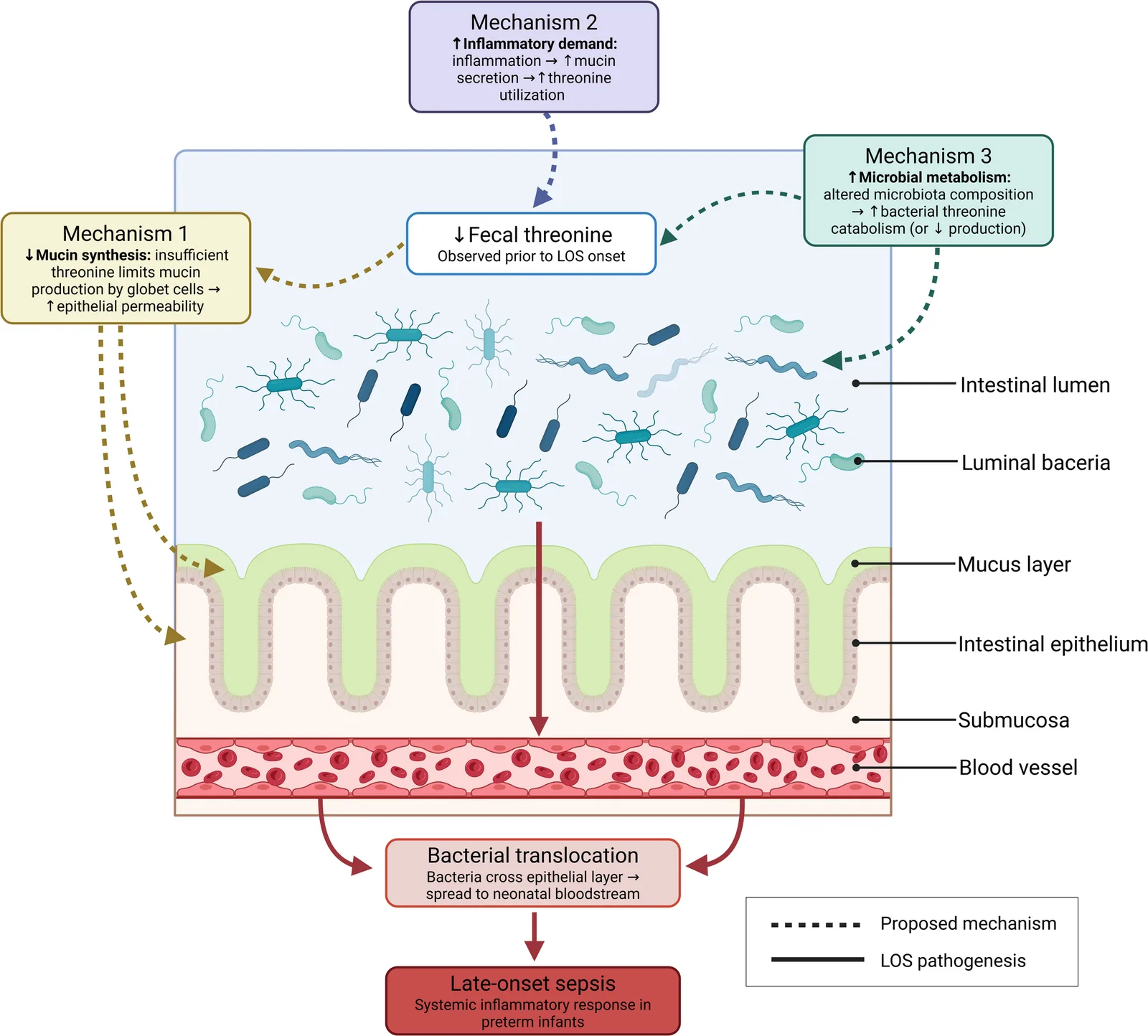
Schematic overview of three proposed mechanisms linking reduced preclinical fecal threonine concentrations to late-onset sepsis in preterm infants. First, insufficient threonine may limit goblet cell mucin synthesis, thinning the protective mucus layer and increasing epithelial permeability. Second, an inflammatory state may transiently upregulate mucin secretion, increasing threonine consumption and thereby lowering fecal levels. Third, altered microbiota composition may enhance bacterial threonine catabolism, reducing luminal availability. These mechanisms may act independently or synergistically, ultimately facilitating bacterial translocation and systemic infection. Future research should elucidate the underlying mechanism of action. Created in BioRender. Amsterdamumc, Eminds (2026) https://BioRender.com/ecfi8ua

The reduction of threonine in both cohorts supports its role in the pathophysiological pathway of LOS and warrants further investigation of the threonine pathway (e.g. addition of end/or intermediary metabolic products). This is supported by recent untargeted metabolomics work, in which the glycine-serine-threonine pathway was identified as a player in LOS pathogenesis (Liu et al., 2024). Additionally, the intestinal uptake of dietary threonine is high in preterm infants (van der Schoor et al., 2007), and luminal threonine is a key substrate for intestinal mucin synthesis, the glycoproteins forming the mucus layer which are critical for maintaining intestinal barrier integrity (Puiman et al., 2011; Schaart et al., 2009; Tang et al., 2021). This provides a biologically plausible mechanism for decreased threonine concentrations in the pathogenesis of LOS. Several potentially interacting mechanisms may underlie this observation (Figure 5). First, insufficient threonine availability may limit mucin synthesis (Mao et al., 2011), compromising the intestinal mucus barrier, increasing epithelial permeability, and facilitating bacterial translocation (Breugelmans et al., 2022; Paone & Cani, 2020). Second, in an inflammatory context, transiently increased mucin secretion (Song et al., 2023) may enhance threonine utilization, resulting lower fecal concentrations. Third, decreased fecal threonine may reflect increased microbial catabolism in the context of altered microbiota composition, as threonine can serve as substrate for bacterial metabolism (Mao et al., 2011). Corresponding alterations in mucin synthesis or turnover are therefore plausible and may serve as additional markers. Other pathways that include threonine, such as microbial fermentation routes that convert threonine into short-chain fatty acids (SCFAs), may also merit investigation. As SCFAs act on epithelial and immune cells to modulate barrier function and inflammation (Parada Venegas et al., 2019), quantifying threonine-derived propionate and butyrate in feces (or their immediate metabolic intermediates) may improve prediction of LOS. Finally, the measurement of L-threonine, used by enterocytes for protein synthesis, and D-threonine, produced through microbial metabolism, instead of their combined concentration may provide additional insights (Roskjær et al., 2024). Studies in other diseases have shown potential for measuring chiral compounds as biomarkers and suggest different biological functions of L- and D-forms of fecal AAs (Liu et al., 2023). Integrating threonine, its chiral forms, and downstream metabolites may improve our understanding of non-staphylococcal LOS pathogenesis and enhance early disease detection.

The current study has several strengths and limitations. The prospective collection of fecal samples allowed identification of preclinical samples, enabling analysis of metabolic changes prior to disease onset. Additionally, the longitudinal design in the matched control infants without LOS and/or NEC provided additional samples to assess postnatal influences, making this the first study, to our knowledge, to do so in the preterm context. Detailed clinical information allowed individual metabolite profiles to be interpreted within their specific clinical context. Both the discovery and validation cohort were analyzed with the same (pre-)analytical methodology, as standardization of preanalytical conditions is essential for obtaining reliable and reproducible results (Opperman et al., 2025). Limitations include the absence of concurrent microbiome data that restricts further mechanistic insight into the observed metabolic patterns. Future studies would benefit from integration of microbiome and metabolome analysis. Additionally, increased sample size allowing pathogen-specific analyses is required to improve understanding of LOS pathogenesis and improve predictive accuracy. Whilst our study included a dedicated panel of 20 AAs including essential, non-essential and conditionally essential AAs, few essential AAs were not included (e.g. arginine, cysteine, aspartic acid). Investigating a broader panel, or using untargeted metabolomics, may yield additional insights. Another limitation is the homogeneity of feeding type within our cohort, which consisted almost exclusively of human milk-fed infants, reflecting standard practice in Dutch NICUs. Given that fecal AA profiles, particularly essential AAs such as threonine, are influenced by dietary intake, findings may not be generalizable to more heterogenous populations, where formula feeding is prevalent. Future studies including formula-fed infants are warranted to elucidate the relationship between diet and fecal AA composition, and potential impact on LOS-associated signals.

In conclusion, our findings provide novel insights into AA-modulating clinical characteristics in the extremely preterm population and the potential role of specific AAs in the pathogenesis and early risk stratification of non-staphylococcal LOS. Although the discriminatory value of threonine was moderate, future studies should evaluate other metabolites within the threonine pathway, or integrate threonine with additional biomarkers to improve understanding and predictive accuracy.

## Supplementary Information

Below is the link to the electronic supplementary material.

Supplementary file1 (DOCX 73 KB)

## Data Availability

The data that support the findings of this study are available on through a Figshare repository: https://doi.org/10.6084/m9.figshare.31123792.
