# Supplementary material for: Longitudinal fecal amino acid profiles in extremely preterm infants: early-life determinants and associations with late-onset sepsis
Source: Metabolomics. 2026 Jun 16;22(4):98. doi: 10.1007/s11306-026-02483-9 (PMC13272250; doi:10.1007/s11306-026-02483-9)
Supplement: Supplementary file 1 — Supplementary file1 (DOCX 73 KB) [file 11306_2026_2483_MOESM1_ESM.docx]

Corresponding manuscript: *Longitudinal fecal amino acid profiles in extremely preterm infants: early-life determinants and associations with late-onset sepsis*

Authors: *A.J. van Wesemael*, R.R. de Kroon*, N.M. Frerichs, M.M. van Weissenbruch, A.H. van Kaam, N.K. de Boer, E. A. Struys, H.J. Niemarkt, T. de Meij, on behalf of the Generation P studygroup*

*Contributed equally to the project

**Supplemental materials**

**Supplemental Table 1.** Chromatographic and mass spectrometric parameters and quantification limits for fecal amino acid analysis by LC-MS/MS.

| **Amino acid** | **LLOQ (μM)** | **Maximum concentration tested within linear range (μM)** | **Compound** | **Polarity** | **Precursor (m/z)** | **Product (m/z)** |
| --- | --- | --- | --- | --- | --- | --- |
| **Glutamine** | 0.5 | 938 | Glutamic acid  Glutamic acid (IS) | Negative  Negative | 368.2  371.2 | 172.1  175.1 |
| **Alfa-amino adipic acid** | 0.05 | 23.3 | Alfa-amino adipic acid  Alfa-amino adipic acid (IS) | Negative  Negative | 382.2  385.3 | 186.1  189.1 |
| **Proline** | 0.5 | 779 | Proline  Proline (IS) | Positive  Positive | 338.2  345.1 | 116.1  123.1 |
| **Glutamine** | 0.5 | 1916 | Glutamine  Glutamine (IS) | Negative  Negative | 367.2  374.2 | 171.1  178.1 |
| **Citrulline** | 0.5 | 247 | Citrulline  Citrulline (IS) | Negative  Negative | 396.2  400.3 | 200.1  204.1 |
| **Serine** | 0.5 | 762 | Serine  Serine (IS) | Negative  Negative | 326.2  329.2 | 130.1  133.1 |
| **Histidine** | 0.5 | 345 | Histidine  Histidine (IS) | Negative  Negative | 376.2  379.2 | 180.1  183.1 |
| **Glycine** | 5 | 1439 | Glycine  Glycine (IS) | Negative  Negative | 296.3  299.3 | 100.0  103.1 |
| **Threonine** | 0.5 | 602 | Threonine  Threonine (IS) | Negative  Negative | 340.2  345.2 | 144.1  149.1 |
| **Phenylalanine** | 0.5 | 739 | Phenylalanine  Phenylalanine (IS) | Negative  Negative | 386.2  391.3 | 190.1  195.1 |
| **Alanine** | 2 | 1437 | Alanine  Alanine (IS) | Negative  Negative | 310.2  314.2 | 114.1  118.1 |
| **Valine** | 0.5 | 726 | Valine  Valine (IS) | Negative  Negative | 338.2  346.2 | 142.1  150.1 |
| **Methionine** | 0.5 | 199 | Methionine  Methionine (IS) | Negative  Negative | 370.2  373.3 | 174.1  177.1 |
| **Leucine** | 0.5 | 725 | Leucine  Leucine (IS) | Negative  Negative | 352.2  355.2 | 156.1  159.1 |
| **Isoleucine** | 0.5 | 436 | Isoleucine  Isoleucine (IS) | Negative  Negative | 352.3  359.2 | 156.1  163.1 |
| **Ornithine** | 0.5 | 496 | Ornithine  Ornithine (IS) | Negative  Negative | 575.2  581.2 | 131.1  137.1 |
| **Tryptophane** | 0.5 | 269 | Tryptophane  Tryptophane (IS) | Negative  Negative | 425.2  430.2 | 229.2  234.1 |
| **Lysine** | 0.5 | 759 | Lysine  Lysine (IS) | Negative  Negative | 589.3  592.3 | 145.1  148.1 |
| *Abbreviations: LC-MS/MS, liquid chromatography tandem mass spectrometry; IS, internal standard; LLOQ, lower limit of quantification; m/z, mass-to-charge ratio; μM, micromolar.* | | | | | | |

**Supplemental Table 2.** Included samples per timepoint, analysis group and disease group

| **Preclinical alterations** | | | | | |
| --- | --- | --- | --- | --- | --- |
| **Discovery cohort** | | | | | |
|  | **T-3** | **T-2** | **T-1** | **T0** | **Total** |
| Non-staphylococcal LOS | 5 | 5 | 6 | 10 | 26 |
| Non-affected matched controls | 5 | 9 | 10 | 10 | 34 |
| *Total* | *10* | *14* | *16* | *20* | *60* |
| **Validation cohort** | | | | | |
| Non-staphylococcal LOS | 5 | 5 | 1 | 2 | 13 |
| Non-affected matched controls | 5 | 7 | 7 | 6 | 25 |
| *Total* | *10* | *12* | *8* | *8* | *38* |
| **Longitudinal analysis** | | | | | |
|  | **Week 1** | **Week 2** | **Week 3** | **Week 4** | **Total** |
| Non-affected matched controls | 12 | 12 | 10 | 8 | 42 |
| In the initial discovery cohort, a total of 93 unique fecal samples is included. In the validation cohort, a total of 38 unique fecal samples is included. | | | | | |

**Supplemental Table 3.** Demographic characteristics of included infants in the validation cohort

|  | **Non-staphylococcal LOS (n=8)** | **Control (n=8)** | **p-value** |
| --- | --- | --- | --- |
| **Gestational age, median (weeks + days), IQR (days)** | 25 + 3 (5) | 25 + 5 (5) | 0.461 |
| **Birth weight (grams), median (IQR)** | 758 (114) | 885 (77) | **0.046** |
| **Biological sex, female, n (%)** | 3 (38) | 5 (63) | 0.617 |
| **Mode of delivery, vaginal delivery, n (%)** | 1 (13) | 3 (38) | 0.564 |
| **Apgar score < 7 at 5 minutes, yes, n (%)** | 1 (13) | 3 (38) | 0.069 |
| **Cumulative days iv antibiotics in first month of life, median (IQR)** | 11 (14) | 6 (3) | **0.035** |
| **Ratio cumulative days iv antibiotics/admission days in first month of life, median (IQR)** | 0.59 (0.41) | 0.19 (0.12) | **0.002** |
| **Cumulative days iv antibiotics prior to onset t-1^1^, median (IQR)** | 5 (3) | 3 (2) | 0.593 |
| **Time to full enteral feeds (days), median (IQR)** | 10 (3) | 8 (3) | 0.737 |
| **Volume feeds (ml/kg/day) at full enteral feeding, median (IQR)** | 156 (8) | 146 (30) | 0746 |
| **Reached full enteral feeding^2^ at t-1, yes, n (%)** | 3 (38) | 4 (50) | 1.00 |
| **Enteral feeding type at t-1:**  -Mother’s own milk, n (%)  -Donor human milk, n (%)  Human milk^3^, n (%) | 6 (75)  0 (0)  0 (0) | 7 (88)  1 (13)  0 (0) | 0.215 |
| **Received enteral probiotics in the first 29 days of life^4^, yes, n (%)** | 7 (87.5) | 7 (87.5) | 1.00 |
| **Cumulative days enteral probiotics in first month of life, median (IQR)** | 19 (20) | 26 (4) | 0.246 |
| **Ratio cumulative days enteral probiotics/admission days in first 29 days of life, median (IQR)** | 0.74 (0.47) | 0.88 (0.17) | 0.371 |
| **Cumulative days enteral probiotics prior to t-1, median (IQR)** | 7 (11) | 6 (10) | 0.712 |
| **Post-natal age (days) at clinical onset of LOS^5^, median (IQR)** | 10 (11.5) | n.a. | n.a. |
| ^1^t-1 is the defined as the day prior to diagnostic work-up that resulted in LOS diagnosis (t0). ^2^Full enteral feeding is defined as the cessation of both parenteral feeding and iv glucose. ^3^Not specified in clinical files whether it was donor human milk or mother’s own milk. ^4^The probiotics mixture administered to the included infants is ProPrems©, according to the Dutch national guidelines. ^5^Clinical onset of LOS is defined as the day the diagnostic work-up is conducted that resulted in LOS diagnosis, which included at least a blood culture. A p-value <0.05 was considered significant. *Abbreviations: IQR, interquartile range; LOS, late-onset sepsis; n.a.: not applicable.* | | | |

**Supplemental Table 4.** Demographic characteristics of LOS infants in discovery and validation cohort

|  | **Discovery LOS (n=12)** | **Validation LOS (n=8)** | **p-value** |
| --- | --- | --- | --- |
| **Gestational age, median (weeks + days), IQR (days)** | 26 + 2 (13) | 25 + 3 (5) | 0.417 |
| **Birth weight (grams), median (IQR)** | 825 (169) | 758 (114) | 0.142 |
| **Biological sex, female, n (%)** | 10 (83) | 3 (38) | 0.104 |
| **Mode of delivery, vaginal delivery, n (%)** | 9 (75) | 1 (13) | **0.022** |
| **Apgar score < 7 at 5 minutes, yes, n (%)** | 1 (8) | 1 (13) | 0.684 |
| **Cumulative days iv antibiotics in first month of life, median (IQR)** | 12 (7) | 11 (14) | 0.757 |
| **Ratio cumulative days iv antibiotics/admission days in first month of life, median (IQR)** | 0.50 (0.37) | 0.59 (0.41) | 0.847 |
| **Cumulative days iv antibiotics prior to onset t-1^1^, median (IQR)** | 3 (3) | 5 (3) | 0.532 |
| **Time to full enteral feeds (days), median (IQR)** | 10 (2) | 10 (3) | 0.811 |
| **Volume feeds (ml/kg/day) at full enteral feeding, median (IQR)** | 159 (6) | 156 (8) | 0.555 |
| **Reached full enteral feeding^2^ at t-1, yes, n (%)** | 6 (50) | 3 (38) | 0.927 |
| **Enteral feeding type at t-1:**  -Mother’s own milk, n (%)  -Donor human milk, n (%)  Human milk^3^, n (%) | 6 (50)  2 (25)  3 (25 | 6 (75)  0 (0)  0 (0) | **0.035** |
| **Received enteral probiotics in the first 29 days of life^4^, yes, n (%)** | 9 (75) | 7 (87.5) | 0.909 |
| **Cumulative days enteral probiotics in first month of life, median (IQR)** | 5 (17) | 19 (20) | 0.361 |
| **Ratio cumulative days enteral probiotics/admission days in first 29 days of life, median (IQR)** | 0.57 (0.52) | 0.74 (0.47) | 0.262 |
| **Cumulative days enteral probiotics prior to t-1, median (IQR)** | 3 (5) | 7 (11) | 0.223 |
| **Post-natal age (days) at clinical onset of LOS^5^, median (IQR)** | 8 (9.5) | 10 (11.5) | 0.847 |
| ^1^t-1 is the defined as the day prior to diagnostic work-up that resulted in LOS diagnosis (t0). ^2^Full enteral feeding is defined as the cessation of both parenteral feeding and iv glucose. ^3^Not specified in clinical files whether it was donor human milk or mother’s own milk. ^4^The probiotics mixture administered to the included infants is ProPrems©, according to the Dutch national guidelines. ^5^Clinical onset of LOS is defined as the day the diagnostic work-up is conducted that resulted in LOS diagnosis, which included at least a blood culture. A p-value <0.05 was considered significant. *Abbreviations: IQR, interquartile range; LOS, late-onset sepsis; n.a.: not applicable.* | | | |

**Supplemental Table 5.** Causative pathogens in discovery and validation cohort

| **Causative pathogen (n, %)** | **Discovery cohort (n=12)** | **Validation cohort (n=8)** |
| --- | --- | --- |
| *Escherichia coli* | 5 (42) | 3 (38) |
| *Streptococcus agalactiae* | 2 (17) | 1 (13) |
| *Enterobacter cloacae* | 1 (8) | 0 (0) |
| *Enterococcus faecalis* | 1 (8) | 1 (13) |
| *Klebsiella* | 1 (8) | 0 (0) |
| *Pseudomonas aeruginosa* | 1 (8) | 0 (0) |
| *Serratia liquefaciens* | 1 (8) | 0 (0) |
| *Serratia marcescens* | 0 (0) | 3 (38) |


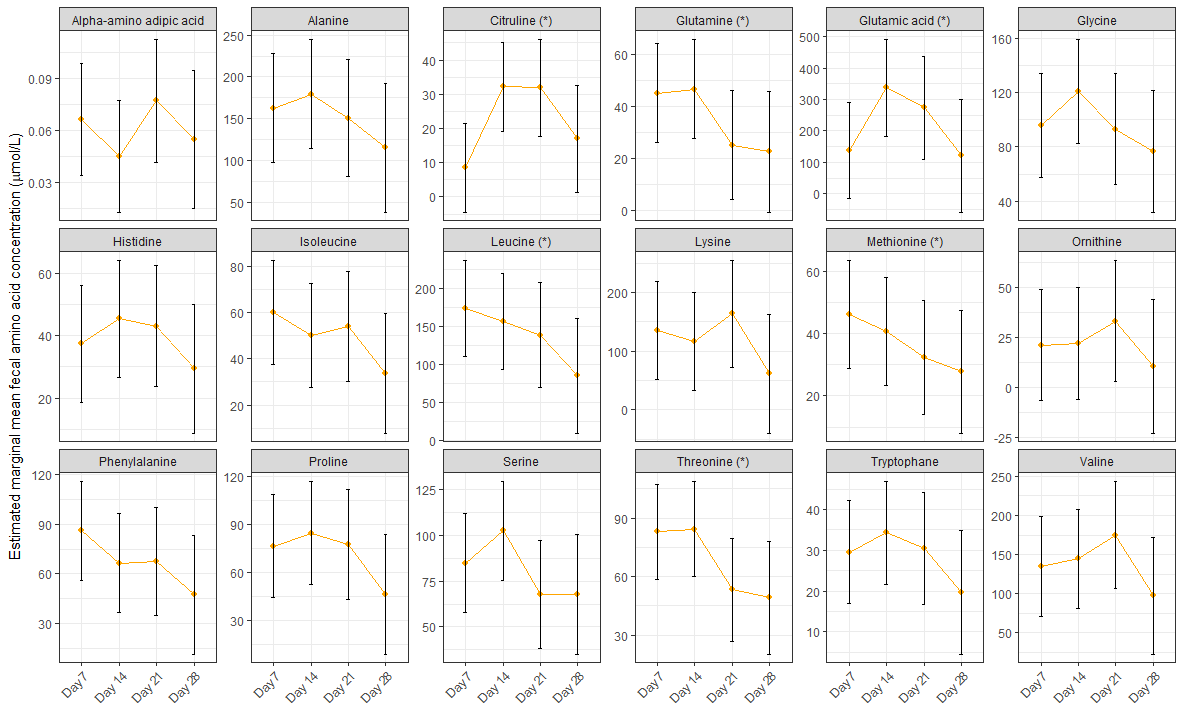


**Supplemental figure S1. Estimated marginal mean amino acid concentrations (μmol/L) in the first month of life in control infants without necrotizing enterocolitis and/or sepsis.** The figure displays the temporal trajectories of the fecal AA concentrations during the first month of life in unaffected matched control infants from the discovery cohort (n = 12 infants), based on model-derived estimates. Each panel depicts one of the measured AAs in infants without NEC and/or sepsis. The y-axis shows the estimated marginal mean AA concentration (μmol/L), and the x-axis shows the postnatal age grouped into sampling windows (day 7±3 days, day 14±3 days, day 21±3 and day 28±3 days). Points represent the estimated marginal means per sampling window derived from linear mixed-effects models, and error bars indicate 95% confidence intervals. Linear mixed-effects models accounting for repeated measurements within control infants (with subject included as a random intercept) were used to evaluate the effect of postnatal age on individual AA concentrations. A p-value <0.05 was considered statistically significant, which is expressed by an asterisk in the header of the plot. *Abbreviations: AA, amino acid; NEC, necrotizing enterocolitis.*
